# CRISPR genotyping as complementary tool for epidemiological surveillance of *Erwinia amylovora* outbreaks

**DOI:** 10.1371/journal.pone.0250280

**Published:** 2021-04-16

**Authors:** Rafael J. Mendes, João Pedro Luz, Conceição Santos, Fernando Tavares

**Affiliations:** 1 Faculty of Sciences of University of Porto, Porto, Portugal; 2 LAQV/REQUIMTE, Faculty of Sciences of University of Porto, Porto, Portugal; 3 CITAB—Centre for the Research and Technology of Agro-Environmental and Biological Sciences, University of Trás-os-Montes e Alto Douro, Vila Real, Portugal; 4 CIBIO–Research Centre in Biodiversity and Genetic Resources, InBIO, Associated Laboratory, University of Porto, Campus Agrário de Vairão, Vairão, Portugal; 5 QRural, Polytechnic Institute of Castelo Branco, School of Agriculture, Castelo Branco, Portugal; Cornell University, UNITED STATES

## Abstract

Fire blight is a destructive plant disease caused by *Erwinia amylovora* affecting pome fruit trees, and responsible for large yield declines, long phytosanitary confinements, and high economic losses. In Portugal, the first major fire blight outbreaks occurred in 2010 and 2011, and although later considered eradicated, the emergence of other outbreaks in recent years stressed the need to characterize the *E*. *amylovora* populations associated with these outbreaks. In this regard, CRISPR genotyping, assessment of three virulence markers, and semi-quantitative virulence bioassays, were carried out to determine the genotype, and assess the virulence of thirty-six *E*. *amylovora* isolates associated with outbreaks occurring between 2010 and 2017 and affecting apple and pear orchards located in the country central-west, known as the main producing region of pome fruits in Portugal. The data gathered reveal that 35 *E*. *amylovora* isolates belong to one of the widely-distributed CRISPR genotypes (5-24-38 / D-a-α) regardless the host species, year and region. Ea 680 was the single isolate revealing a new CRISPR genotype due to a novel CR2 spacer located closer to the leader sequence and therefore thought to be recently acquired. Regarding pathogenicity, although dot-blot hybridization assays showed the presence of key virulence factors, namely *hrpL* (T3SS), *hrpN* (T3E) and *amsG* from the amylovoran biosynthesis operon in all *E*. *amylovora* isolates studied, pathogenicity bioassays on immature pear slices allowed to distinguish four virulence levels, with most of the isolates revealing an intermediate to severe virulence phenotype. Regardless the clonal population structure of the *E*. *amylovora* associated to the outbreaks occurring in Portugal between 2010 and 2017, the different virulence phenotypes, suggests that *E*. *amylovora* may have been *introduced* at different instances into the country. This is the first study regarding *E*. *amylovora* in Portugal, and it discloses a novel CRISPR genotype for this bacterium.

## 1. Introduction

*Erwinia amylovora*, an Erwiniaceae bacterium species [1], is the etiological agent of fire blight, a destructive plant disease that affects the productive potential of trees, and the entire pome fruit trees production sector, of enormous value in Portugal, belonging particularly to the *Amygdaloideae* subfamily, previously named *Spiraeoideae* [2] of the Rosaceae family [3, 4]. *E*. *amylovora* susceptive fruit tree species include major commercial species like pear (*Pyrus communis*.), apple (*Malus domestica*), quince (*Cydonia oblonga*) and loquat (*Eriobotrya japonica*), some ornamental species, and wild species [3, 5–8]. The symptoms of fire blight manifest in the form of blighted shoots, trunks, leaves and roots, which usually become brownish, leading the infected hosts to develop a burnt aspect, as if they were consumed by fire [4]. *E*. *amylovora* enters through natural openings and wounds in host plants, colonizing the intercellular spaces, migrating into the vascular system, and producing biofilms that block xylem vessels inhibiting water transport. This excessive biofilm production can lead to originate bacterial exudates [9–12]. These exudates are acknowledged as major sources of *E*. *amylovora* dissemination to other nearby hosts through wind, water, insects and by cultural practices, namely pruning using contaminated tools [11, 13].

The lack of efficient phytosanitary measures to control and prevent fire blight outbreaks, which is essential to prevent the dissemination of this disease, has led to the classification of *E*. *amylovora* as a quarantine species, ranked in the list of the ten most important bacterial plant pathogens alongside other phytopathogens [14–16]. A major challenge faced by phytosanitary authorities concerning fire blight is the difficulty to timely detect the entry of *E*. *amylovora* in a new area/country, and to implement scientifically informed containment measures [7].

Fire blight originates from North America and has spread to numerous countries in different continents, namely Europe, Oceania and Western Asia. The disease was first reported in Europe in 1957 in England, and has rapidly spread to other European countries, such as France, Spain, Serbia, Croatia, Tunisia, Hungary, and Switzerland [17–22]. This worldwide distribution of *E*. *amylovora* genotypes may have been particularly favored by the commerce of infected rootstocks and saplings of host plants, on which the disease passes easily unnoticed, as the bacteria can live as an endophyte or epiphyte [23, 24].

Comprehensive epidemiological surveys based on non-sequencing typing molecular approaches namely, ribotyping, random amplified polymorphic DNA fragment (RAPD) [25–27], amplified fragment length polymorphism (AFLP) [26, 28, 29], amplified ribosomal DNA restriction enzyme analysis (ARDREA) [30], pulsed field gel electrophoresis (PFGE) [23, 26, 31, 32], variable number of tandem repeats (VNTR) [33], and comparative genomics analysis studies [34–37], reveal a low genetic and genomic diversity within the *E*. *amylovora Amygdaloideae*-infecting strains.

This is likely explained by low recombination and a narrow host-range, evoking a specialization, which is further supported by several pseudogenes and genome reduction of genes, particularly associated with energy metabolism [38]. Regardless this genomic homogeneity, efforts have been made to disclose *E*. *amylovora* population structure and to chronologically trace back the origin of fire blight outbreaks [7, 33, 38–41], in order to identify the emergence of bacterial lineages associated to new outbreaks and unveil their dispersion patterns, which is important to advise phytosanitary authorities about the most suitable measures for containment of fire blight and evaluate their efficacy.

The clustered regularly interspaced short palindromic repeat sequences (CRISPR) and the CRISPR-associated (Cas) proteins, known as the CRISPR-Cas system, integrate short DNA sequences, designated as spacers (usually between 28 and 34 bp), namely from bacteriophages, plasmids, or other laterally-transferred DNA sequences, in a temporal sequence manner and separated by direct repeats (DR), with the first acquired spacer located at the 3’ end of each array and the latest spacer at the 5’ end, i.e. close to the leader sequence [as reviewed by 42–44]. These features make CRISPR a fast-evolving loci in bacteria, providing a fine-tune genotyping method to assess the genetic diversity of bacteria characterized by a high clonality and, most importantly, a chronological record with previous encounters with heterogeneous DNA, and in a less extent with endogenous DNA (self-target), which is particularly useful for epidemiological source tracking [as reviewed by 42, 43, and 44]. In fact, CRISPR genotyping has been widely used to assess the population structure of several Enterobacteriaceae human pathogens, such as *Escherichia coli*, *Yersinia pestis*, and *Salmonella enterica* [42, 45–47], but also of plant pathogenic *E*. *amylovora* clones [21, 38–40, 48, 49]. The works of McGhee and Sundin [40], and Rezzonico *et al*. [39] have set up the bases of a CRISPR genotyping scheme for *E*. *amylovora* by the identification and characterization of three CRISPR arrays in *E*. *amylovora*, namely CRISPR1 (CR1), CRISPR2 (CR2), and CRISPR3 or 4 (CR3 or 4), which allowed to discriminate several *E*. *amylovora* genotypes and identify the most prevalent lineages in different world regions.

Previous studies of comparative genomics, CRISPR and MLVA genotyping, have grouped *E*. *amylovora* in four different clades, all with the same epidemiological origin (North America), and mainly distinguished by their host preference (*Amygdaloideae*- or *Rubus*-infecting strains), supporting the high clonality observed within the species [33, 35, 37, 39–41]. Other studies have shown that *E*. *amylovora* strains display different levels of virulence [7–8, 36, 50–52].

Regardless these efforts, direct assessment by hybridization-based methods of genetic determinants of pathogenicity and virulence in *E*. *amylovora*, namely of genes coding for the type III secretion system (T3SS), genes coding for the corresponding effector proteins (T3E), largely acknowledge as essential to modulate the plant defense system in several pathosystems [38, 53], and genes from the exopolysaccharide (EPS) amylovoran biosynthesis operon, described as a major virulence factor for *E*. *amylovora* (as reviewed by Pique et al., 2015 [38]), have only been scarcely reported. Furthermore, studies have shown that dot-blot hybridization assays under high stringency conditions, are particularly suitable to specifically detect several diagnostic and virulence markers simultaneously [54, 55].

Portugal, where *E*. *amylovora* is considered by EPPO to be present but under eradication, the first indisputable evidence of fire blight outbreak occurred in 2010, affecting several apple and pear orchards in the main production regions of the country [56]. Since then, several reoccurring outbreaks have been reported [56], and despite the major impact that fire blight might have in Portuguese agribusiness, attending the 257 kha of apples and pears producing area [57], there is no knowledge about the *E*. *amylovora* lineages responsible for these outbreaks, neither about their origin, nor virulence.

This work aimed to characterize the population of *E*. *amylovora* associated to fire blight outbreaks occurring in Portugal from 2010 until 2017, through CRISPR genotyping complemented by dot blot hybridization detection of three virulence markers and immature pear slice bioassays to determine symptoms severity. The data gathered showed a clonal population within which distinct virulence phenotypes could be observed. Furthermore, the finding of a new spacer leading to a new CR2 pattern, may suggest the appearance of a new evolving *E*. *amylovora* genotype. These results call for the need of attentive phytosanitary surveillance, particularly of the higher virulent strains.

## 2. Materials and methods

### 2.1. Bacterial isolates and culture conditions

The 36 *E*. *amylovora* isolates used in this study were isolated between 2010 and 2017 from fruits, trunk exudates, and branches from symptomatic apple and pear orchards trees, located at the main production region of pome fruits in Portugal (Table 1, S1 Fig), and were cultured at 28°C on King’s B medium (20 g of Peptone protease number 3; 10 mL of glycerol; 1.5 g of K_2_HPO_4_; 1.5 g of MgSO_4_.7H_2_O; 18 g of agar; distilled water up to 1L) [58]. Bacterial cultures were stored in King’s B medium at -80°C in 30% glycerol. In the present study, *E*. *amylovora* isolates and the type strain LMG 2024 (used as a positive control for polymerase chain reaction amplification (PCR), dot blot hybridization and immature pear assay), were recovered and cultured on King’s B medium at 28°C.

**Table 1:** Portuguese *Erwinia amylovora* isolates used in this work.

Strain	Host		Isolated from	Geographic origin	Year
	Species	Cultivar			
**Ea 230**	Pear	Rocha	Exudate	Alcobaça	2010
**Ea 240**	Pear	Rocha	Exudate	Alcobaça	2010
**Ea 250**	Pear	Rocha	Branch	Alcobaça	2010
**Ea 260**	Pear	Rocha	Branch	Alcobaça	2010
**Ea 270**	Pear	Passe Crassane	Branch	Alcobaça	2010
**Ea 280**	Pear	Rocha	Exudate	Alcobaça	2011
**Ea 310**	Pear	Rocha	Exudate	Alcobaça	2011
**Ea 320**	Pear	Rocha	Branch	Alcobaça	2011
**Ea 340**	Pear	Rocha	Branch	Alcobaça	2011
**Ea 350**	Pear	Rocha	Branch	Alcobaça	2011
**Ea 390**	Apple	Royal Gala	Necrotic fruit	Alcobaça	2011
**Ea 410**	Apple	Royal Gala	Semi-necrotic fruit	Alcobaça	2011
**Ea 430**	Apple	Royal Gala	Semi-necrotic fruit	Alcobaça	2011
**Ea 450**	Pear	Rocha	Exudate	Alenquer	2015
**Ea 460**	Pear	Rocha	Exudate	Alenquer	2015
**Ea 470**	Pear	Rocha	Exudate	Alenquer	2015
**Ea 480**	Pear	Rocha	Exudate	Alenquer	2015
**Ea 490**	Pear	Rocha	Branch	Alenquer	2015
**Ea 500**	Pear	Rocha	Branch	Alenquer	2015
**Ea 510**	Pear	Rocha	Exudate	Alenquer	2015
**Ea 520**	Pear	Rocha	Exudate	Alenquer	2015
**Ea 540**	Pear	Carapinheira	Branch	Caldas da Rainha	2015
**Ea 570**	Pear	Carapinheira	Branch	Caldas da Rainha	2015
**Ea 580**	Pear	Carapinheira	Branch	Caldas da Rainha	2015
**Ea 610**	Apple	Gala	Branch	Cadaval	2015
**Ea 620**	Apple	Gala	Branch	Cadaval	2015
**Ea 630**	Apple	Gala	Branch	Cadaval	2015
**Ea 670**	Pear	Rocha	Branch	Cadaval	2015
**Ea 680**	Pear	Rocha	Branch	Cadaval	2015
**Ea 720**	Pear	Rocha	Branch	Cadaval	2015
**Ea 730**	Pear	Unidentified	Branch	West*	2017
**Ea 740**	Pear	Unidentified	Branch	West*	2017
**Ea 750**	Pear	Unidentified	Branch	West*	2017
**Ea 780**	Pear	Unidentified	Branch	West*	2017
**Ea 790**	Pear	Unidentified	Branch	West*	2017
**Ea 820**	Pear	Unidentified	Branch	West*	2017

* These isolates have been isolated in the West region of Portugal, which includes the municipalities Alcobaça, Caldas da Rainha, Alenquer and Cadaval (S1 Fig).

### 2.2. DNA extraction and quantification

For molecular diagnosis, CRISPR genotyping, and dot blot hybridization, genomic DNA was obtained for all isolates from pure cultures using the E.Z.N.A.^®^ Bacterial DNA Kit (Omega Bio-Tek, Carlsbad, CA, USA) following the manufacturer’s instructions. DNA quantification was carried out using the Qubit^®^ 2.0 Fluorometer (Thermo Fisher Scientific, Waltham, Massachusetts, USA).

### 2.3. *Erwinia amylovora* PCR identification

Identification of the isolates as *E*. *amylovora* was carried out by PCR following the standard diagnostic protocols for *E*. *amylovora* as recommended by EPPO [59], using three pairs of chromosomal specific [60–62], and one pair of pEA29 specific primers [63] (Table 2). PCR reactions were carried out using a reaction mixture containing 1x DreamTaq Buffer with 2.0 mM MgCl_2_ (Thermo Fisher Scientific, Waltham, Massachusetts, USA), 0.2 mM of dNTPs (GRiSP, Porto, Portugal), 0.2 μM of each primer, 1U of DreamTaq DNA Polymerase (Thermo Fisher Scientific, Waltham, Massachusetts, USA), and 25 ng of DNA template. PCR cycling conditions for the chromosomal specific primers (G1-F+G2-R, FER1-F+FER1-R, and FER1-F+rgER2R) and for the plasmid specific primers (PEANT1+PEANT2) were the same as detailed by the EPPO standard diagnostic protocol [59]. PCR products were separated by electrophoresis in a 0.8% agarose gel stained with GreenSafe Premium (NZYTech, Lisbon, Portugal), with constant voltage (90V) in 1x Tris-EDTA (TE) buffer. The agarose gel was observed with a GelDoc™ (Bio-Rad Laboratories, California, USA).

**Table 2:** List of primers used in this work.

Primer	Sequence (5’-3’)	Reference	Purpose
**G1-F**	CCTGCATAAATCACCGCTGACAGCTCAATG	[60]	PCR identification
**G2-R**	GCTACCACTGATCGCTCGAATCAAATCGGC	[60]	PCR identification
**FER1-F**	AGCAGCAATTAATGGCAAGTATAGTCA	[61]	PCR identification
**FER1-R**	AATTTAATCAGGTCACCTCTGTTCAAC	[61]	PCR identification
**rgER2R**	AAAAGAGACATCTGGATTCAGACAAT	[62]	PCR identification
**PEANT1**	TATCCCTAAAAACCTCAGTGC	[63]	PCR identification
**PEANT2**	GCAACCTTGTGCCCTTTA	[63]	PCR identification
**CR1-F1**	CGCCGCCACGCTGCCATTT	[40]	CR1 amplification
**C1-R0**	TCCAGCGCCTGTAAAGCGGC	[40]	CR1 amplification
**CR1RevRpt**	CGGTTTATCCCCGCTCACGC	[40]	CR1 amplification
**CR1-F1-G1**	CAAGCGACAACCTGTTTTTCAGT	This work	CR1 amplification
**CR1-F1-G1-2_0**	ACTGAAATTTAAAATCACCGCTAA	This work	CR1 amplification
**CR1-F1-G1-3_0**	CTATGCAGAAGCGGAGGG	This work	CR1 amplification
**CR1-F1-G1-4_0**	CAAGCGATCAACCTTTTT	This work	CR1 amplification
**CR1-F1-G2**	TCTCATCCCTCATGTTTTCCA	This work	CR1 amplification
**C1-R0-G1**	AGCAGTACGTTGACTGTAAA	This work	CR1 amplification
**C1-R0-G1-2_0**	AAGAACGTCAACAATTGCATT	This work	CR1 amplification
**C1-R0-G2**	TTGGCGGAGAGGATTTTACAAT	This work	CR1 amplification
**C1-R0-G3**	TTTCAGTGCTCATGCTCATGCGCAAT	This work	CR1 amplification
**CR3-F1**	TTTTCGCCGGGACAG	[40]	CR3 amplification
**CR3-R1**	AAGACCGGAAGCAAAGTA	[40]	CR3 amplification
**CR2-F1**	GCGGCCAACAGATGCGGAAAAG	[40]	CR2 amplification
**CR2-R1**	TGCGGGGAACACTCGACATCTAAT	[40]	CR2 amplification
**CR2-F2**	GTCTGGCGCAAAAACTGGAG	[40]	CR2 amplification
**CR2-F3**	CCGCCCTTCTGGTGTTTTGA	[40]	CR2 amplification
**CR2-R2**	ACACGTGGTTTCTGAGTCTGGA	[40]	CR2 amplification
**hrpL-F**	GCTTAATATTGATTGGGAAGGC	This work	Dot-blot assay
**hrpL-R**	ACCAGCATGTTCAACAGACG	[64]	Dot-blot assay
**hrpN-F**	AATGCAAAGCCTGTTTGGTG	This work	Dot-blot assay
**hrpN-R**	CCATGAACTGACCGATTTCC	[64]	Dot-blot assay
**amsG-F**	GCTTTATGGCACGGATATGG	[64]	Dot-blot assay
**amsG-R**	GAGTAATACGGGGGTCG	This work	Dot-blot assay

### 2.4. Assessing pathogenicity by dot blot hybridization

Pathogenicity potential of the 36 *E*. *amylovora* isolates were evaluated by dot blot hybridization of three virulence markers designated by *hrpN*^M^, *hrpL*^M^, and *amsG*^M^, targeting respectively the *hrpL* gene which is a transcriptional switch of the T3SS *hrp* operon [38, 65]; the *hrpN* gene coding for a translocator protein [66, 67]; and *amsG*, a gene coding the amylovoran biosynthesis protein AmsG, involved in the *ams* gene cluster [38, 68].

DNA probes for dot blot hybridization were prepared by PCR amplification of the three virulence specific markers on *E*. *amylovora* type strain LMG 2024, using primers previously described by Pester *et al*. [64] for each gene, and by designing their respective complementary primers (Table 2), resorting to the full genome of the type strain LMG 2024 (AN: CAPB00000000.1), to obtain DNA probes with the following sizes: 368bp for *amsG*^M^, 378bp for *hrpL*^M^, and 410bp for *hrpN*^M^. A 50 μL PCR reaction mix consisted of 1x DreamTaq Buffer with 2.0 mM MgCl_2_ (Thermo Fisher Scientific, Waltham, Massachusetts, USA), 0.2 mM of dNTPs (GRiSP, Porto, Portugal), 0.5 μM of each forward and reverse primer, 1.25U of DreamTaq DNA Polymerase (Thermo Fisher Scientific, Waltham, Massachusetts, USA), and 10 ng of DNA template. For negative control, H_2_O was added to the reaction mix instead of DNA template. PCR cycle parameters were carried out with a first amplification cycle of 3 min at 95°C, followed by 30 cycles at 95°C for 30 s, 50°C for 30 s, and 72°C for 60 s, and a final extension at 72°C for 5 min. The obtained PCR products were purified using the illustra GFX™ PCR DNA and Gel Band Purification Kit (GE Healthcare, Chicago, Illinois, USA), and sequenced (STAB Vida, Caparica, Lisbon, Portugal) to confirm the identity of each amplicon used as probe. Probes were labelled with digoxigenin using the DIG-High Prime kit, according to the manufacturer’s instructions (Roche Diagnostics, Basel, Switzerland). The dot blot hybridization with labelled probes was carried out using 100 ng bacterial DNA from the 36 *E*. *amylovora* isolates, that were spotted onto nylon membranes (Roche Diagnostics, Basel, Switzerland) using a Bio-Dot apparatus (Bio-Rad, Hercules, California, USA). Each membrane was hybridized overnight at 68°C to ensure high stringency with a final probe concentration of 100 ng/mL, and stringency washes were performed following manufacturers recommendation. Probe-target hybrids were detected with chemiluminescent alkaline phosphatase substrate (CDP-Star®) reagent (Roche Diagnostics, Basel, Switzerland) and the images were acquired using a Molecular Imager ChemiDoc™ system (Bio-Rad, Hercules, California, USA). Three dot blot hybridization replicates for each probe were carried out.

### 2.5. CRISPR amplification and sequencing

Complete CR1, CR2, and CR3 arrays were sequenced for the 36 *E*. *amylovora* isolates, using primers detailed in Table 2. The PCR cycling parameters were 5 min at 94°C, followed by 40 cycles of 94°C for 30 s, 58°C or 55°C (CR1/2, and CR3 respectively) for 30 s, 72°C for 4 min or 45 s (CR1/2, and CR3 respectively), and a final extension at 72°C for 7 min. PCR reactions were established in a final volume of 50 μL containing 1x DreamTaq Buffer with 2.0 mM MgCl_2_ (Thermo Fisher Scientific, Waltham, Massachusetts, USA), 0.2 mM of dNTPs (GRiSP, Porto, Portugal), 0.4 μM of each primer, 1U of DreamTaq DNA Polymerase (Thermo Fisher Scientific, Waltham, Massachusetts, USA), and 10 ng of DNA template. PCR amplicons were separated by electrophoresis in a 0.8% agarose gel stained with GreenSafe Premium (NZYTech, Lisbon, Portugal), at a constant voltage (90V) in 1x TE buffer, and purified using the Illustra GFX™ PCR DNA and Gel Band Purification Kit (GE Healthcare, Chicago, Illinois, USA) following manufacturer’s instructions. Sequencing of the three CRISPR arrays was outsourced to STAB Vida (Costa da Caparica, Lisbon, Portugal), and primer walking sequencing was employed to obtain the full sequence of the CR1/2 arrays.

### 2.6. CRISPR array analysis

Raw CR1/2 and 3 array sequences were assembled using the Geneious program version 11 (Biomatters, Auckland, New Zealand) and Benchling Life Sciences RandD Cloud (Benchling, San Fransisco, USA). CRISPR array spacers and DRs patterns were generated with resource to CRISPRs finder tool (https://crispr.i2bc.paris-saclay.fr/). Both spacers and DRs of the CR1/2 and 3 arrays were further assessed by BLASTn for existing homology in GenBank database (http://blast.ncbi.nlm.nih.gov/Blast.cgi). To identify novel spacers and patterns, the CRISPR array patterns of the 36 *E*. *amylovora* isolates, together with the *E*. *amylovora* type strain LMG 2024, and the North American reference strain ATCC 49946, were compared with CRISPR array patterns previously described [39, 40, 49].

### 2.7. Nucleotide sequences accession numbers

DNA sequences corresponding to the three CRISPR regions (CR1, CR2, and CR3) of the 36 *E*. *amylovora* Portuguese isolates were deposited in the National Center for Biotechnology Information (NCBI) database with the following AN: MK778646 to MK778682 for CR1; MK784021 to MK784056; and MN402458 for CR2; and MK764044 to MK764080 for CR3 (S1 Table).

### 2.8. Virulence assays in immature pear slices

Pathogenicity assays were conducted as described by EPPO [59] on slices of immature pear fruits (*P*. *communis* cv. General Leclerc), previously surface sterilized with 70% ethanol. Briefly, 10 μL of bacterial suspensions of colonies of each *E*. *amylovora* isolate (Table 1) were inoculated on stab wounded slices (three 5 mm superficial wounds per slice), at a concentration of 10^9^ cfu.mL^-1^ in PBS (10 mM, pH 7.2), followed by an incubation at 25°C during 7 days under high relative humidity conditions. Results were considered positive when necrotic lesions and bacterial ooze appeared on the injured tissue. Photographic records of the symptoms were made, and the virulence of each isolate was evaluated seven days after inoculation, using an ordinal categorical scale of damage (0 to 6) adapted from Schwarczinger *et al*. [69], namely, 0 –symptomless; 1 –low exudate production with browning in ¼ of the slice; 2 –production of exudate with browning in half of the slice; 3 –production of exudate with light browning of the slice; 4 –production of exudate with dark browning of the slice; 5 –enhanced production of exudate with intense dark browning of the slice; 6 –scorched slice. Analysis of damage was made using a blind evaluation to avoid bias errors and ensue impartiality. All pathogenicity assays were repeated in three independent experiments with two biological replicates and three technical replicates per experiment and inoculated isolate (i.e., two pear slices of different pear fruit and three wounds per slice were used for each experiment and inoculated isolate). PBS was used as negative control, and type strain LMG 2024 as positive control. The data is reported as the frequency values of the different damage categories observed for the 36 *E*. *amylovora* isolates.

## 3. Results

### 3.1. *Erwinia amylovora* PCR identification

**Fig 1:**
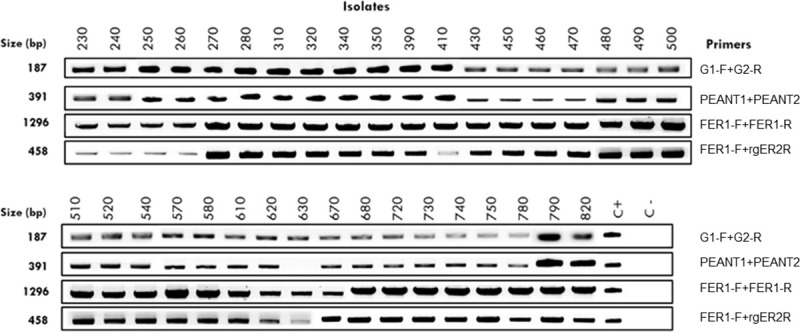
Identification of *Erwinia amylovora* Portuguese isolates by PCR, using specific primer pairs, according to EPPO protocol [59]. Bacterial isolates are identified by strain numbers on the top. Primers used are listed on the right, and DNA product sizes in bp are indicated on the left. Positive control (C+): Type Strain LMG 2024. Negative control (C-): H_2_O.

The 36 bacterial isolates were identified as *E*. *amylovora*, using four species-specific DNA markers recommended by EPPO for diagnosis [59]. For the 36 bacterial isolates tested, all four markers were amplified with product sizes corresponding to the expected length of the markers (187, 391, 458, and 1296 bp, for G1-F+G2-R, PEANT1+PEANT2, FER1-F+rgER2R, and FER1-F+FER1-R, respectively), except for the isolate Ea 630, for which no amplification was obtained with primers PEANT1+PEANT2, targeting the plasmid pEA29 specific marker (Fig 1). The type strain LMG 2024 was used as positive control for the four markers, confirming the expected length size of each marker, whilst no amplification was obtained for the negative control whatever the marker.

### 3.2. Pathogenicity evaluation by dot blot hybridization

**Fig 2:**
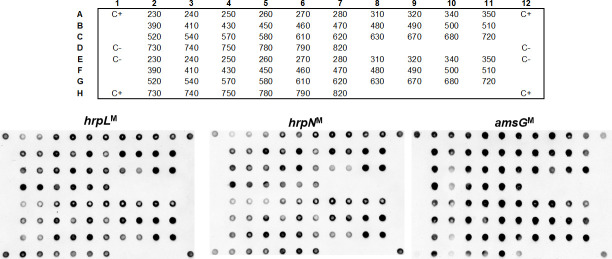
Dot blot results. Dot blot using three probes (*hrpL*^M^, *hrpN*^M^ and *amsG*^M^) and genomic DNA from 36 *Erwinia amylovora* isolates. The top grid represents the position of the DNA from each *E*. *amylovora* isolate in the nylon membrane. Positive control (C+): Type Strain LMG 2024. Negative control (C-): TE Buffer.

To investigate the putative pathogenicity, and to further confirm the identity of the 36 *E*. *amylovora* isolates, three genes known to be important for *E*. *amylovora* infection, namely *hrpL*, *hrpN*, and *amsG*, were assessed by dot blot hybridization. Consistent hybridization dots were observed for the three virulence markers (*hrpN*^M^, *hrpL*^M^, and *amsG*^M^) for all the 36 *E*. *amylovora* isolates studied (Fig 2), further confirming the identification of these isolates as *E*. *amylovora*.

### 3.3. CRISPR spacers array patterns/genotype and profile

CRISPR array diversity was analyzed using the CRISPRs finder tool, for the 36 *E*. *amylovora* bacteria isolated between 2010 and 2017 from Portuguese outbreaks affecting apple and pear orchards (Table 1, S1 Fig). *E*. *amylovora* type strain LMG 2024 was used for comparison. DRs of the three *E*. *amylovora* CRISPR array systems were shown to be identical to what has been described previously [39]. In fact, DRs of CR1/2 array patterns have a length of 29 nt each, and show a high similarity differing in two nt, namely, a guanine instead of an adenine, and an adenine instead of a thymine in positions 14 and 15, respectively, of CR1 DR. The CR3 array pattern has a DR with 28 nt, and showed no similarity with the CR1/2 DRs.

CRISPR genotyping led to the identification of 75 unique spacers within the set of the 37 *E*. *amylovora* strains used in this study (i.e., the 36 *E*. *amylovora* isolates, plus the type strain LMG 2024, Fig 3, S2 Table). From these 75 unique spacers identified, 74 have been previously characterized [39, 40], and a novel CR2 spacer (NS) was identified and located next to the leader sequence in Ea 680. This spacer, composed of 32 nt (NS, 5’–TGTATGGCATATTGCGGGCGGGTGCTTGTCAT– 3’), lacked similarity to other spacers of the CRISPR database (https://crispr.i2bc.paris-saclay.fr/crispr/BLAST/CRISPRsBlast.php). Interestingly, BLASTn analysis of this new spacer allowed to find 100% identity with an intergenic genomic region located between a putative Major Facilitator Superfamily (MFS) fosmidomycin resistance (Fsr) transporter and Kef family K(+) transporter of 10 *E*. *amylovora*, and 90.62% identity to *Erwinia pyrifoliae* Ejp617 (Table 3).

**Fig 3:**
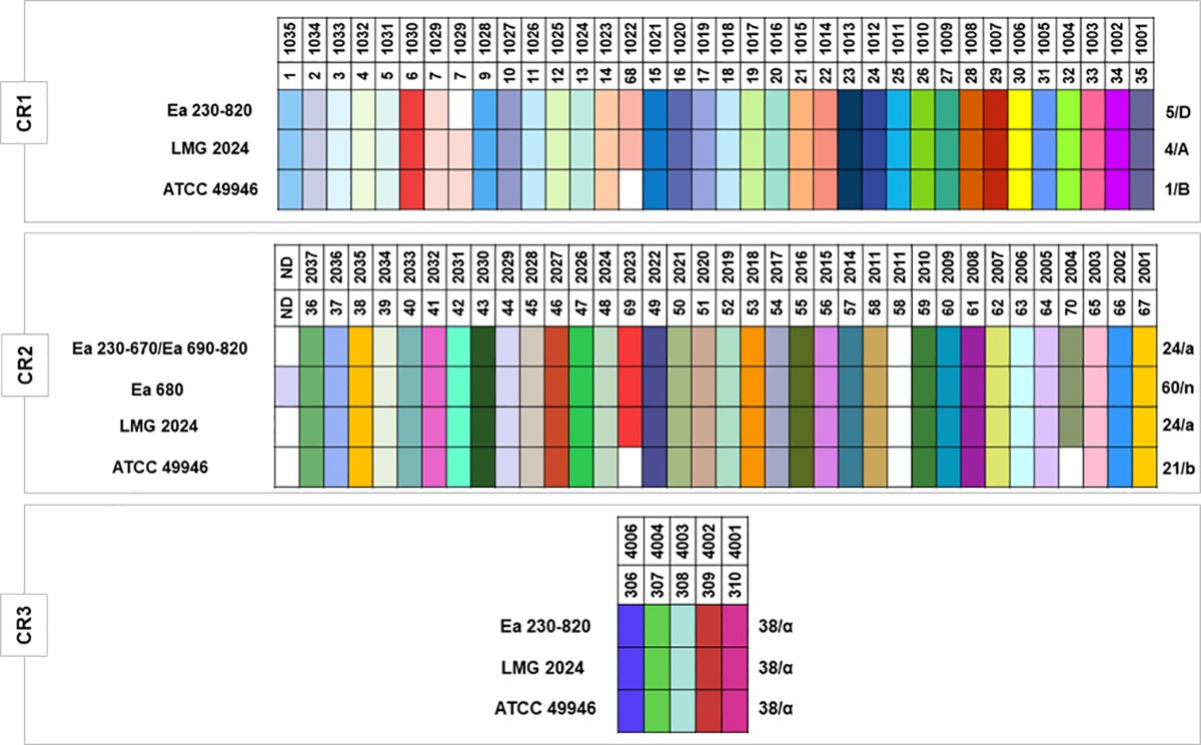
CRISPR arrays patterns of the 36 Portuguese *Erwinia amylovora* isolates and type strain LMG 2024. CR1/2 and 3 array patterns for the strains ATCC 49946, were retrieved from McGhee and Sundin [40] and Rezzonico et al. [39]. Isolates identification are listed on the left of each color pattern. Each spacer is represented by a single color for each CRISPR array. Spacers were considered unique if they contained at least 5 nucleotide differences compared to other spacers. Each spacer is identified by a color and a number in the upper side of each CR. Spacers that are equal are aligned in column for the different isolates in study. The blank intervals represent a spacer that it is not present in that CR pattern. Patterns/genotypes are identified by a number and a letter respectively in the right side of the color pattern. Nomenclature of each spacer, and pattern/genotype were based on the classification of McGhee and Sundin [40], and Rezzonico et al. [39], respectively.

**Table 3:** BLASTn results of the nucleotide sequence of the novel spacer.

Strain	Query Cover	E Value	Identity	Accession number	Sequence Range	Origin
*Erwinia amylovora* strain FB-20	100%	7e-07	100%	CP050240.1	2662948–2662979	South Korea
*Erwinia amylovora* strain FB-86	100%	7e-07	100%	CP050258.1	2663421–2663452	South Korea
*Erwinia amylovora* strain FB-207	100%	7e-07	100%	CP050263.1	2663421–2663452	South Korea
*Erwinia amylovora* strain FB-307	100%	7e-07	100%	CP050242.1	2663299–2663330	South Korea
*Erwinia amylovora* strain TS3238	100%	7e-07	100%	CP050244.1	2663420–2663451	South Korea
*Erwinia amylovora* strain E-2	100%	7e-07	100%	CP024970.1	1143072–1143103	Belarus
*Erwinia amylovora* strain TS3128	100%	7e-07	100%	CP056034.1	1109120–1109151	South Korea
*Erwinia amylovora* IL-5	100%	7e-07	100%	FR719189.1	44473–44504	USA
*E*. *amylovora* CFBP1430	100%	7e-07	100%	FN434113.1	1106308–1106339	France
*E*. *amylovora* ATCC 49946	100%	7e-07	100%	FN666575.1	1143796–1143827	USA
*Erwinia pyrifoliae* Ejp617	100%	0.035	90.62%	CP002124.1	2369652–2369683	Japan

The results showed that CR3 array patterns, or genotypes observed for the 37 *E*. *amylovora* strains have been previously described [39, 40]. CR3 array pattern consisting of 5 spacers, corresponds to pattern 38 as defined by McGhee and Sundin [40], or genotype “α” as determined by Rezzonico *et al*. [39], for the 37 *E*. *amylovora* strains. Concerning the CR1 array patterns that consists of 35 spacers, whilst the 36 Portuguese *E*. *amylovora* isolates correspond to pattern 5, or genotype “D”, the type strain LMG 2024 presented pattern 4, or genotype “A” as previously reported by both of the mentioned studies (Fig 3). Regarding CR2, two patterns/genotypes were identified for the Portuguese isolates, namely pattern 24, or genotype “a”, consisting of 34 spacers observed for 35 *E*. *amylovora* Portuguese isolates (Ea 230–670 and Ea 690–820), identical for the *E*. *amylovora* type strain LMG 2024, and a new pattern/genotype discerned for strain Ea 680 consisting of 35 spacers and originated by the inclusion of a novel spacer (NS), herein designated as pattern 60, or genotype “n”.

When the three CRISPR array patterns were combined, although the majority of the 37 *E*. *amylovora* bacteria studied could be distributed in one common CRISPR profile (5-24-38 / D-a-α), the *E*. *amylovora* Portuguese isolate Ea 680 revealed a new profile (5-60-38 / D-n-α) (Table 4). Even though these results disclose a high clonality for the Portuguese isolates (35 presented the same profile), the new CRISPR profile observed indicates a putative new trend of *E*. *amylovora* diversity.

**Table 4:** CRISPR array profile/genotype found in *Erwinia amylovora* isolates.

CRISPR array profile^a^/genotype^b^ (CR1-CR2-CR3)	Number of profile/genotype *E*. *amylovora* isolates found	Regions previously found
5-24-38 / D-a-α	35 Portuguese	Western U.S. / Europe / Middle East / New Zealand
5-60-38 / D-n-α	1 Portuguese (Ea 680)	First reported in Portugal / Newly reported in this work

^a^ as described by McGhee and Sundin [40]

^b^ as decribed by Rezzonico *et al*. [39]

### 3.4. Pathogenicity bioassays and levels of virulence

**Fig 4:**
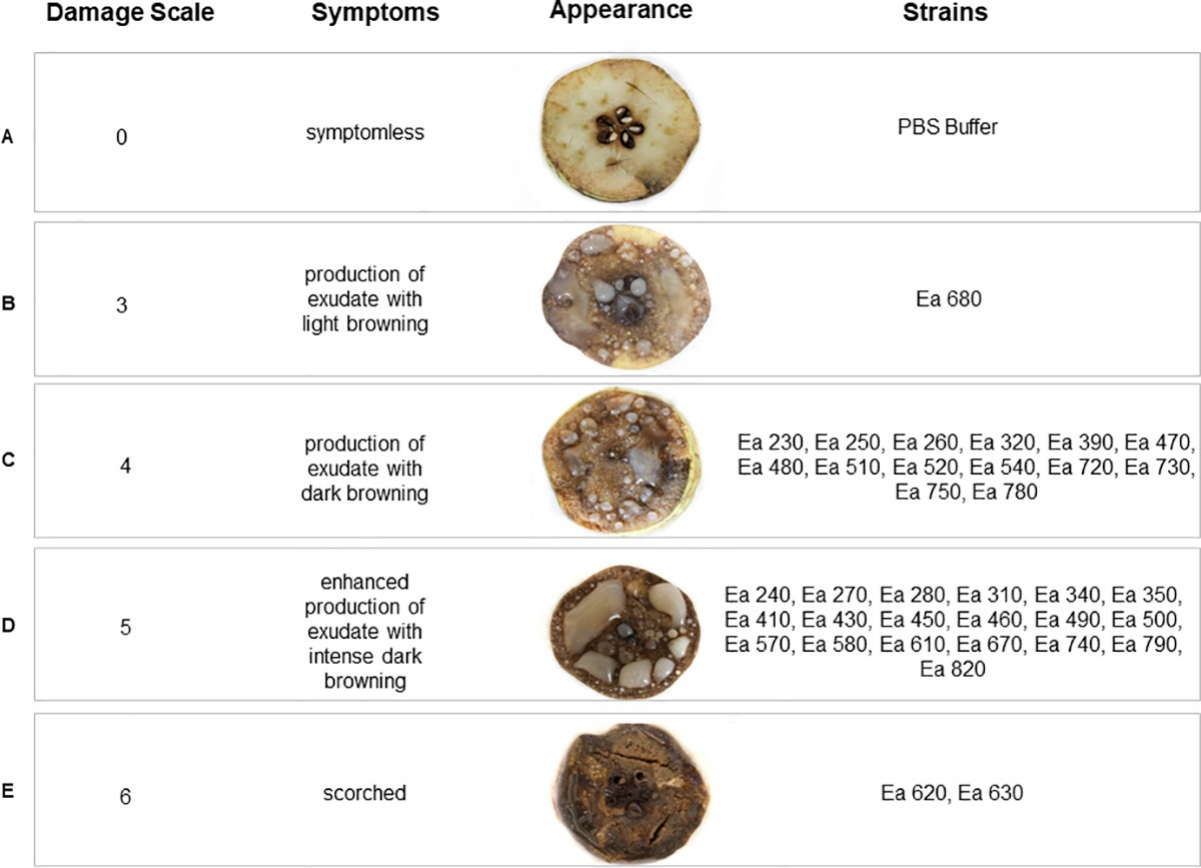
Virulence assay results. Symptoms observed in immature pear slices 7 days after infection with *Erwinia amylovora* isolates, were categorized according a damage scale adapted from Schwarczinger et al. [69]. Positive control (C+): Type Strain LMG 2024. Negative control (C-): PBS Buffer.

Pathogenicity was demonstrated for the 36 *E*. *amylovora* Portuguese isolates through inoculation of 10^9^ cfu.mL^-1^ of each strain in immature pear slices. When assessing symptom severity using a damage scale, it was possible to observe four distinct levels of virulence (Fig 4), ranging between scale values 3 and 6. Fourteen isolates (38.8%) produced exudates and caused dark browning of the pear slice (scale value 4, Fig 4C), and 19 isolates (52.8%) produced enhanced exudate and caused intense dark browning of the pear slice (scale value 5, Fig 4D). The remaining three isolates were observed as outliers, with isolate Ea 680 (2.8%) causing exudates with light browning (scale value 3, Fig 4B), and isolates Ea 620 and Ea 630 (5.6%) scorching the pear slice (scale value 6, Fig 4E). Taking into account epidemiological relevant data of the 36 *E*. *amylovora* isolates, namely year of isolation, geographic origin, host species or host cultivars (Table 1, S1 Fig), no evidence for a connection with the virulence symptoms was found.

## 4. Discussion

Fire blight disease caused by *E*. *amylovora* continues to be a major concern in pome fruit producing countries, including Portugal, where severe outbreaks have been reported since 2010, causing high economic damage in pear and apple orchards, located on the country’s central-west, known as the main producing region. Although the *E*. *amylovora* isolates associated to these outbreaks have been cryopreserved, no data about its genetic diversity, pathogenicity, and virulence were available, impairing an epidemiological characterization and inferences about the origin of these outbreaks.

In the present study, following the identification of these isolates as *E*. *amylovora*, we proceeded with CRISPR genotyping and pathogenicity bioassays in order to unveil the population structure of *E*. *amylovora* associated to these outbreaks. Conventional PCR using four *E*. *amylovora* specific pair of primers, targeting for three chromosomal markers [60–62], and a pEA29 plasmid marker [63], as recommended by EPPO [59], has shown specific amplification for the four markers for all the isolates, with exception of the pEA29 plasmid marker for strain Ea 630. However, this result does not weaken the identification as *E*. *amylovora*, since previous studies provided evidence that some *E*. *amylovora* wild-type strains cured for plasmid pEA29 did not compromised its pathogenicity potential [70, 71].

To further confirm the identity of the 36 *E*. *amylovora* Portuguese isolates and concomitantly address their pathogenicity potential, *the presence of essential virulence-related genes namely, hrpL [38, 65], hrpN [65, 66], and amsG* [38, 68] were investigated by dot blot hybridization. Although dot blot using species-specific markers has not been used as a diagnostic tool for *E*. *amylovora*, it has been used as a fine-tuned technique for diagnoses of a broad range of plant and animal pathogens, capable to overcome the limitations of PCR-based methods, particularly the occurrence of false-negatives due to primers’ mismatches [55, 72, 73]. In addition, dot blots allow the simultaneous screening of numerous samples and may conciliate both taxonomic and functional markers, which may contribute to determine different typing features [74]. These results acknowledged that the virulence related genes used for dot blot hybridization, are suitable molecular markers for *E*. *amylovora*, as shown by the positive hybridization signals obtained for all screened isolates, suggesting that all isolates hold a pathogenicity potential and are positive for *amsG*, which is a signature marker for *E*. *amylovora*. Furthermore, dot blot hybridization assays consistently supported PCR-based *E*. *amylovora* identification using primer pairs targeting a gene coding for glucosyltransferase-I precursor (G1-F+G2R), two hypothetical protein coding loci as predicted by Glimmer/Critica (FER1-F+FER2-R, FER1-F+rgER2R), and one intergenic region (PEANT1+PEANT2) [59]. These data confirm that the isolates associated to Portuguese fire blight outbreaks between 2010 and 2017 were indeed *E*. *amylovora*.

In order to disclose the genetic diversity of these isolates and to make possible a comprehensive genotyping assessment with a worldwide collection of *E*. *amylovora* strains, CRISPR loci were sequenced for all the 36 isolates. CR3 array, acknowledged as the shortest and most conserved CRISPR locus in *E*. *amylovora*, showed the presence of five spacers revealing pattern 38, according to McGhee and Sundin [40] or genotype “α” according to Rezzonico *et al*. [39] nomenclature. This pattern/genotype was identical to what has been extensively described for most *Amygdaloideae*-infecting *E*. *amylovora* strains characterized so far, therefore holding poor discriminatory resolution.

Regarding CR1 locus, it was possible to identify pattern 5 or genotype “D”, for all the 36 Portuguese isolates which were shown to miss the duplication of spacer 7/1029, differing from the type strain LMG 2024 that contains the mentioned duplication, and from the representative North American strain ATCC 49946, which lacks the spacer 68/1022. This CR1 pattern/genotype has been reported to occur commonly in other European, Middle East, New Zealand, and Western U.S. *Amygdaloideae*-infecting *E*. *amylovora* strains [40].

Pattern 24, or genotype “a”, was observed for CR2 locus in 35 out of the 36 Portuguese *E*. *amylovora* isolates used in this study. This pattern/genotype is identical to what was reported for type strain LMG 2024 and differ from the North American strain ATCC 49946 by the presence of spacers 69/2023 and 70/2004, similarly to what has been found in several *Amygdaloideae*-infecting *E*. *amylovora* strains with a broad distribution, namely in North America, Europe, Middle East, and New Zealand [40]. Interestingly, isolate Ea 680 was shown to differ from the other 35 *E*. *amylovora* isolates by the presence of a novel spacer (NS) in CR2 locus next to the leader sequence. This NS that has not been reported so far, discloses a novel CR2 pattern/genotype, herein designated 60/”n”, according to the nomenclature of McGhee and Sundin [40] and Rezzonico *et al*. [39], respectively. The high identity of the NS to non-coding regions located outside the CRISPR loci of ten *E*. *amylovora* strains and of *E*. *pyrifoliae* Ejp617, as shown by BLASTn analysis, further confirms its CR2 spacer signature. This may be due to a recent acquisition derived from foreign genetic elements or from its own genome as a self-target, as has been described to occur in some bacteria [43]. In addition, and with the exception for *E*. *amylovora* strain FB-306, which showed a new CR2 pattern/genotype due to the loss of two spacers (54/2017 and 55/2016), the CR2 patterns/genotypes for the other nine *E*. *amylovora* strains, namely CR2 pattern 22 and 24, have been acknowledged to occur in other *Amygdaloideae*-infecting *E*. *amylovora* [40, 49]. Regarding the *E*. *pyrifoliae* Ejp617, isolated from *Rubus* in Japan, the NS was not present in the CRISPR loci of this strain, although the DRs of CR2 are identical to CR2 of *Amygdaloideae*-infecting *E*. *amylovora*, which is in line with what has been previously described for the *Rubus*-infecting *E*. *amylovora* IL-5 strain [40].

When the CR1, 2, and 3 loci are combined, the CRISPR profile observed for the 35 Portuguese *E*. *amylovora* isolates (5-24-38 / D-a-α) was identical to strains from Europe, namely Ea 322, UPN 527, ACW 64132, ACW 63230; from Middle East, namely LebA-3 and LebA-18, isolated from pear trees in Lebanon; from New Zealand, namely NZR3, NZR5, and NZS24; and to strains from Western U.S., namely 87–73 and 87–70 [39, 40]. This profile is aligned with the global distribution of this genotype and suggests a high clonality but unknown origin for the *E*. *amylovora* isolates associated to Portuguese outbreaks reported in Portugal from 2010 to 2017. The new CRISPR profile 5-60-38/D-n-α observed in the Portuguese isolate Ea 680, does not refute the high clonality of the Portuguese *E*. *amylovora* population neither a distinct origin from the other 35 isolates, since it was due to the likely recent acquisition of a single new spacer by CR2 and, therefore, can be used as a strain-specific marker to investigate the dispersion and prevalence of this strain in new outbreaks. These results are aligned with comparative genomics studies [35–37]. In fact, when comparing the profiles of the 35 *E*. *amylovora* Portuguese isolates with the strains described in these genomics studies, they fit in the ‘Widely-Prevalent’ clade, which is broadly distributed around the world, as mentioned above.

CRISPR has been used as a resourceful genotyping tool to disclose *E*. *amylovora* population structure, and has already been hypothesized that it can affect pathogenicity and virulence to some extent in *E*. *amylovora* [75]. However, it is inefficient to inform about pathogenicity and virulence of *E*. *amylovora*, attending that genetic determinants of pathogenicity and virulence are located in distinct genomic regions (e.g., plasmids; pathogenicity genomic islands). Therefore, these determinants are exposed to distinct selective pressures and consequently to distinct evolutionary dynamics as emphasized by comprehensive comparative and functional genomics studies (as reviewed by Llop *et al*., [76]; Llop, [77]; Yuan *et al*., [78]). Despite genetic differences between *E*. *amylovora* strains showing distinct virulence phenotypes being reported, the genomic landscape responsible for a higher or lower virulence is still unknown as emphasized previously [36]. Accordingly, pathogenicity and virulence phenotypic characterization are still the most robust approaches to distinguish *E*. *amylovora* strains regarding strain-specific fitness to cause disease and for risk assessment analysis [36]. Pathogenicity bioassays carried out in immature pear slices coupled with a scale to measure symptoms’ severity to determine distinct levels of virulence allowed to confirm that all the 36 Portuguese *E*. *amylovora* isolates were pathogenic but showed four distinct virulence phenotypes. While most of the isolates revealed to be considerably virulent (scale value 4 and 5), two isolates showed the most severe virulence phenotype (scale value 6: Ea 620 and Ea 630), and only a single isolate resulted in a milder virulence phenotype (scale value 3: Ea 680). Interestingly, it has been suggested that the low virulence in a strain can be caused by a single nucleotide polymorphism in the *hfq* gene, that is responsible of encoding a small RNA chaperone [36] or due to the lack of the plasmid pEI70 [71]. On the other hand, other studies suggest that higher virulence is generally linked to a higher gene expression of the *amsG* gene encoding for amylovoran [79]. Interestingly these three isolates (Ea 620, Ea 630, and Ea 680) isolated in 2015 from pears and apples at the same location (Cadaval), showed distinct virulence phenotypes, which may suggest different origins worth being further investigated by whole genome sequencing.

## 5. Conclusion

In this work we showed that the fire blight outbreaks affecting the main pear and apple production area in Portugal between 2010 and 2017 were caused by a highly clonal population of *E*. *amylovora* similar to a well-represented genotype distributed worldwide, namely present in Europe, New Zealand, Middle-East, and Western U.S., as evaluated by CRISPR genotyping. Regardless this low genomic diversity, the work allowed to disclose a new CRISPR genotype due to a new CR2 spacer, which sequence shows full similarity to intergenic regions of other *E*. *amylovora*, suggesting an episode of self-target acquisition.

Dot blot hybridization analysis, besides further confirming the identity of *E*. *amylovora* isolates, suggested that all the 36 Portuguese isolates were pathogenic. To unveil distinct virulence behaviours among the Portuguese isolates, pathogenicity tests in pear slices, combined with a scale to categorize symptoms damage, revealed four distinct virulence phenotypes, which could not be attributed to host species, year of isolation, or geographic origin.

Taken together, the results call for the need to identify virulence expression markers that may resolve the genotypic homogeneity of *E*. *amylovora* isolates as revealed by CRISPR or other sequence-based genotyping methods.

## Supporting information

S1 Fig Map of main producing regions of Portugal. Main apple and pear producing regions in Portugal from where the isolates (Ea 230–580), were obtained (orange markers). Map downloaded from Natural Earth (naturalearthdata.com) and edited in QGIS v3.16.0 (qgis.org).
(TIF)

S1 Table Isolates used in this work. List of *Erwinia amylovora* isolates collected in Portugal, and type strain LMG 2024, used in this study and the correspondent GenBank accession number of CR1, 2, and 3 regions sequences.
(DOCX)

S2 Table Nucleotide sequence of individual CRISPR spacers identified in this study. (DOCX)
