# Supplementary figures and images for: CRISPR genotyping as complementary tool for epidemiological surveillance of Erwinia amylovora outbreaks
Source: PLoS One. 2021 Apr 16;16(4):e0250280. doi: 10.1371/journal.pone.0250280 (PMC8051791; doi:10.1371/journal.pone.0250280)

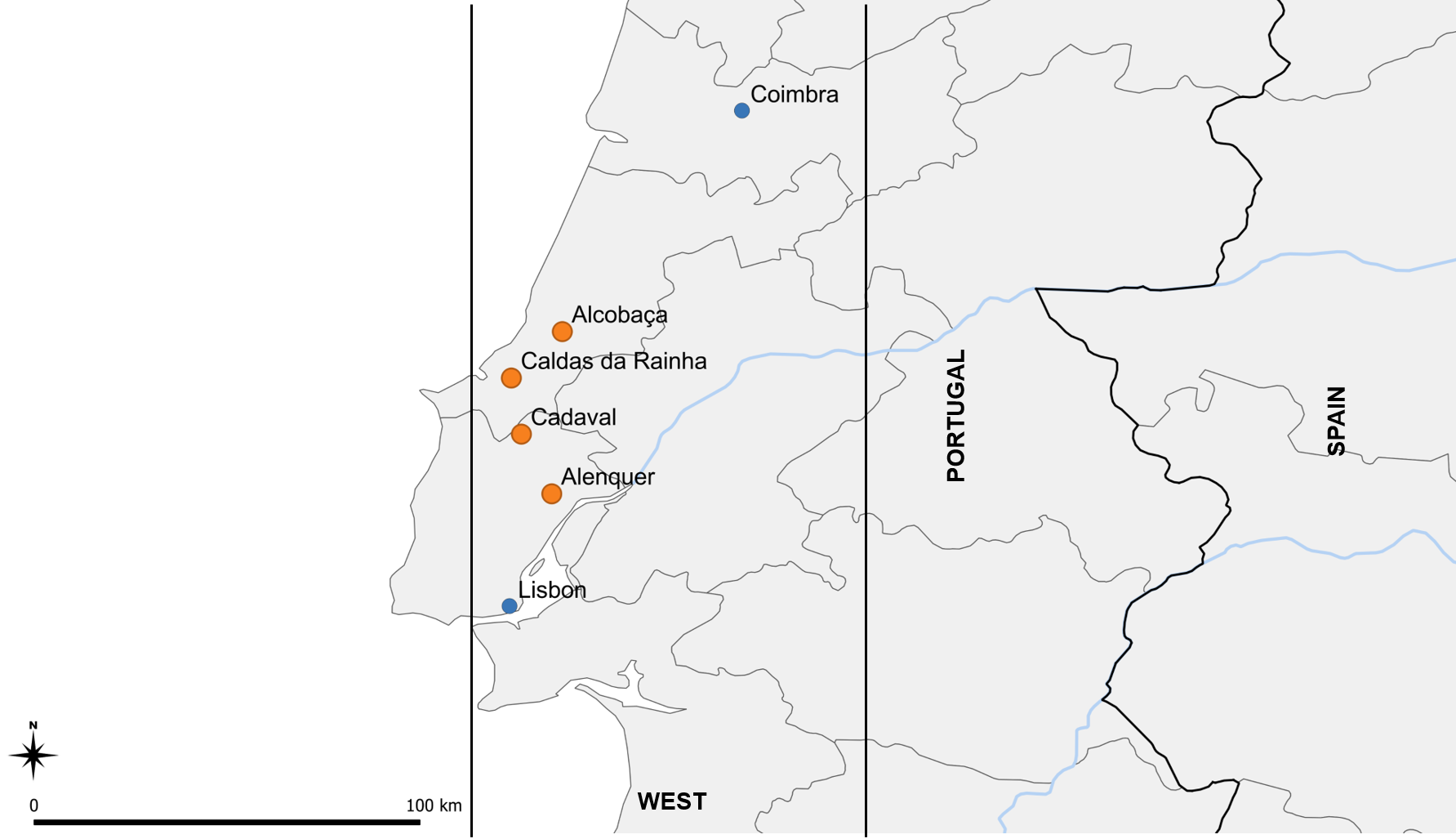

Supplement: S1 Fig — Main apple and pear producing regions in Portugal from where the isolates (Ea 230–580), were obtained (orange markers). Map downloaded from Natural Earth (naturalearthdata.com) and edited in QGIS v3.16.0 (qgis.org). (TIF) [file pone.0250280.s001.tif]
