# Supplementary material for: CRISPR genotyping as complementary tool for epidemiological surveillance of Erwinia amylovora outbreaks
Source: PLoS One. 2021 Apr 16;16(4):e0250280. doi: 10.1371/journal.pone.0250280 (PMC8051791; doi:10.1371/journal.pone.0250280)
Supplement: S1 Table — List of Erwinia amylovora isolates collected in Portugal, and type strain LMG 2024, used in this study and the correspondent GenBank accession number of CR1, 2, and 3 regions sequences. (DOCX) [file pone.0250280.s002.docx]

| Isolate | CR1 Accession Number | CR2 Accession Number | CR3 Accession Number |
| --- | --- | --- | --- |
| Ea 230 | MK778646 | MK784021 | MK764044 |
| Ea 240 | MK778647 | MK784022 | MK764045 |
| Ea 250 | MK778648 | MK784023 | MK764046 |
| Ea 260 | MK778649 | MK784024 | MK764047 |
| Ea 270 | MK778650 | MK784025 | MK764048 |
| Ea 280 | MK778651 | MK784026 | MK764049 |
| Ea 310 | MK778652 | MK784027 | MK764050 |
| Ea 320 | MK778653 | MK784028 | MK764051 |
| Ea 340 | MK778654 | MK784029 | MK764052 |
| Ea 350 | MK778655 | MK784030 | MK764053 |
| Ea 390 | MK778656 | MK784031 | MK764054 |
| Ea 410 | MK778657 | MK784032 | MK764055 |
| Ea 430 | MK778658 | MK784033 | MK764056 |
| Ea 450 | MK778659 | MK784034 | MK764057 |
| Ea 460 | MK778660 | MK784035 | MK764058 |
| Ea 470 | MK778661 | MK784036 | MK764059 |
| Ea 480 | MK778662 | MK784037 | MK764060 |
| Ea 490 | MK778663 | MK784038 | MK764061 |
| Ea 500 | MK778664 | MK784039 | MK764062 |
| Ea 510 | MK778665 | MK784040 | MK764063 |
| Ea 520 | MK778666 | MK784041 | MK764064 |
| Ea 540 | MK778667 | MK784042 | MK764065 |
| Ea 570 | MK778668 | MK784043 | MK764066 |
| Ea 580 | MK778669 | MK784044 | MK764067 |
| Ea 610 | MK778670 | MK784045 | MK764068 |
| Ea 620 | MK778671 | MK784046 | MK764069 |
| Ea 630 | MK778672 | MK784047 | MK764070 |
| Ea 670 | MK778673 | MK784048 | MK764071 |
| Ea 680 | MK778674 | MN402458 | MK764072 |
| Ea 720 | MK778675 | MK784049 | MK764073 |
| Ea 730 | MK778676 | MK784050 | MK764074 |
| Ea 740 | MK778677 | MK784051 | MK764075 |
| Ea 750 | MK778678 | MK784052 | MK764076 |
| Ea 780 | MK778679 | MK784053 | MK764077 |
| Ea 790 | MK778680 | MK784054 | MK764078 |
| Ea 820 | MK778681 | MK784055 | MK764079 |
| LMG 2024 | MK778682 | MK784056 | MK764080 |
