# Supplementary material for: CRISPR genotyping as complementary tool for epidemiological surveillance of Erwinia amylovora outbreaks
Source: PLoS One. 2021 Apr 16;16(4):e0250280. doi: 10.1371/journal.pone.0250280 (PMC8051791; doi:10.1371/journal.pone.0250280)
Supplement: S2 Table — (DOCX) [file pone.0250280.s003.docx]

| Number (McGhee and Sundin, [38]) | Number (Rezzonico *et al*., [37]) | Sequence (5’ – 3’) |
| --- | --- | --- |
| 1 | 1035 | TTATTCATGAGCCTTTTTATCTTCGCGGCATG |
| 2 | 1034 | GTAAATAGCAAAATGATAAATAATTTATCAAT |
| 3 | 1033 | CTATGCAGAAGCGGAGGGCGGCGAGTGATGGA |
| 4 | 1032 | AGCATCTCGGGAACTGTGTTTTTTGTATAAAA |
| 5 | 1031 | AAGATGCTTTGACATTAATTATCTCCATAAAA |
| 6 | 1030 | CAAGCGATCAACCTGTTTTTCAGTAGGTTTAA |
| 7 | 1029 | GATTGCGCATGAGCACTGAAATTGTTCACAGC |
| 9 | 1028 | ACAAAAGACAACACCCCCCTTACCCCCCCACG |
| 10 | 1027 | CAGGTATTTCGGATAGCCGGTTGTCTCGGCGG |
| 11 | 1026 | ACTGAAATTTAAAATCACCGCTAACCCGCCAG |
| 12 | 1025 | GGCGATGAGGGAGTACGCGGAGCGGCAGGGTA |
| 13 | 1024 | AAAAGCCAACCGCCCGCCCGTAATAAACCTGA |
| 14 | 1023 | GTTGCAGAGACTTAAAGATCGTCTGCTAGTTA |
| 15 | 1021 | TAAAGGAGCATGCTTATACAACTGACAAAATC |
| 16 | 1020 | AGATTTGGCGGAAATGTCGGCGGAGATGCCCC |
| 17 | 1019 | AAATGTCCTGTGGCTCGGCCCGATGCTGCAAT |
| 18 | 1018 | GAGATCATTCTCATCCCTCATGTTTTCCAGGA |
| 19 | 1017 | ATTGTAAAATCCTCTCCGCCAAATTTGATTAC |
| 20 | 1016 | AAACTCTCGCATACATGGACGGAATTTAACGA |
| 21 | 1015 | ACGATTTGCCTGAAACCTCAACGAAGTTCGAC |
| 22 | 1014 | CTGATGGCGTCACGAGCCATACGGAATGTGAC |
| 23 | 1013 | CAAAAATTTGCGCATGTCATCTATCTTTTTTT |
| 24 | 1012 | CCCTCGGGGAGGGCTTTGCGTTGTTACTCAGA |
| 25 | 1011 | GTTACGTTGAATGTATCGTTGGATGTGATTAA |
| 26 | 1010 | TACATCGAACAATGCCAATTGTTGACGTTCTT |
| 27 | 1009 | CCGCGAAAATCCGCAGTGAGCTGGCAATGAGC |
| 28 | 1008 | GCTGTCTATCTGGGCTGCCTCTATCCAGCAAT |
| 29 | 1007 | ACTTCGGTGAGAATGTCGAATTGCCACCAGAT |
| 30 | 1006 | TTGAATCAGAGTCTTTCAGGGACGATGTTTTC |
| 31 | 1005 | TGAAGCAGCCAGAATCCCATCCGGCCTTTATC |
| 32 | 1004 | GCTTTTGTACCCTTTACAGTCAACGTACTGCT |
| 33 | 1003 | AGATTGAGATCTTATCAACGGACTCTGACGCC |
| 34 | 1002 | TTTCTTCACACACAACGGTGAGGGCATTGTCT |
| 35 | 1001 | TGATAAAGTAACGTTCCGAATGGCGCGTGATG |
| 36 | 2037 | CCATTTTATGACAGTCTGGCGCAAAAACTGGA |
| 37 | 2036 | GAGATGCACTGGATATACCGACTCCTCACTGA |
| 38 | 2035 | GCTCGGGGGGACATGAGCTTGTACAAAACAGC |
| 39 | 2034 | TTTTTAGCAGCGTGACAGTTATGGAGCCGCTC |
| 40 | 2033 | ATTATAAGGATCACTTGCTAGGGCATTATATA |
| 41 | 2032 | ACTTGACTGTTTATGCAGTGGTTGTATTTCTT |
| 42 | 2031 | ATCGGAACGACTTAGATTAGCGTCCTTGCACAT |
| 43 | 2030 | TAACCGCAACCATCGCCGCGATAAATCCACTG |
| 44 | 2029 | GTTGATACGGCTGATTACAATAAAATGTCACT |
| 45 | 2028 | TAAAAAATGCCGCCATCGAATCAGCAAAATCG |
| 46 | 2027 | CTGCGGAGCGTCAAACGGGCGTTAACTCTCGA |
| 47 | 2026 | CCCTTCTGGTGTTTTGATTCTCCTAGGTGATT |
| 48 | 2024 | AATGGACGAGATTTCACAGAAAATATCTGTTC |
| 49 | 2022 | CAGATGAGGCTGCAAATTCCAGGCACTTTTTA |
| 50 | 2021 | GATGGTCGTACCGATGTTTGCGAAAGATTCGC |
| 51 | 2020 | ACGGTCAGATGGTGGCGCTGGTTGCGCTGGCA |
| 52 | 2019 | CTGTTTATGAAAAATGCCAACAAACAGGAAGC |
| 53 | 2018 | ATTTTTCAGGAACGGGCCGACACGAAAATTTAT |
| 54 | 2017 | ATATTTACTAGCATTTCCCCATGCTGTATCAC |
| 55 | 2016 | CTGGAGCATGAGACGAAATCGGGGGTAGTGCT |
| 56 | 2015 | CCGGTTCAGGTTTGATAGGTTCTGCCTAACTC |
| 57 | 2014 | TTCGCATACGACAATCTCCCGGCACTGATTAA |
| 58 | 2011 | AGGGTGACGCAACGATTGTTGCAATTCCTAAC |
| 59 | 2010 | CACCAGTGTGTACATTCCAGACTCAGAAACCAC |
| 60 | 2009 | CCTCGAGGTGTTCTAAGCACTCCGGGGCTTTT |
| 61 | 2008 | GACGCTCAAATCAGTGGCGGCGAAACCCGACA |
| 62 | 2007 | CCAGAGGGGATTTAGCAAACGTCATTTCTGAC |
| 63 | 2006 | TCATCTGCGGGTCGGGTAGGCTGCTTACGGGT |
| 64 | 2005 | CAGCTATTCCCCGCATCGGTCAGTACTGCGCT |
| 65 | 2003 | GCCAATGGATTCAGGATTGGAGCCAGAATTTA |
| 66 | 2002 | AAAAAAAGCCTAAAGCTCGAAAGAATAAAAAT |
| 67 | 2001 | ATGATGGCGCTGATAGTTTTATTAGATGTCGA |
| 68 | 1028 | TAAATGGTTGTCCGTTCTTGGCGCAGACGGCT |
| 69 | 2023 | GCTACTACGTGTACGCACAGCCGCTGGCCAGT |
| 70 | 2004 | TGGCCCACAATGGTAAAACCGGCGGCTTTCCA |
| 306 | 4006 | TTTACGTTTGCGTTAACAGTAAGCTCTGCAAC |
| 307 | 4004 | ATCGCACCCCACTGATTGAAGAGCAGCACACT |
| 308 | 4003 | TAGCAATAAATTCGATAGACGCTGATTTGCGT |
| 309 | 4002 | TGGCTTCAATTGCGGTCGGGTGTGATGCATCA |
| 310 | 4001 | GCCAACGTTCACTGTCATTTAGCCACGCTTCCG |
| NS | NS | TGTATGGCATATTGCGGGCGGGTGCTTGTCAT |
